# A novel *Pseudomonas aeruginosa* Bacteriophage, Ab31, a Chimera Formed from Temperate Phage PAJU2 and *P. putida* Lytic Phage AF: Characteristics and Mechanism of Bacterial Resistance

**DOI:** 10.1371/journal.pone.0093777

**Published:** 2014-04-03

**Authors:** Libera Latino, Christiane Essoh, Yann Blouin, Hoang Vu Thien, Christine Pourcel

**Affiliations:** 1 Univ Paris-Sud, Institut de Génétique et Microbiologie, UMR 8621, Orsay, France; 2 CNRS, Orsay, France; 3 Hôpital Armand Trousseau, Assistance Publique-Hôpitaux de Paris (APHP), Bactériologie, Paris, France; Centro Nacional de Biotecnologia - CSIC, Spain

## Abstract

A novel temperate bacteriophage of *Pseudomonas aeruginosa,* phage vB_PaeP_Tr60_Ab31 (alias Ab31) is described. Its genome is composed of structural genes related to those of lytic *P. putida* phage AF, and regulatory genes similar to those of temperate phage PAJU2. The virion structure resembles that of phage AF and other lytic *Podoviridae* (*S. enterica* Epsilon 15 and *E. coli* phiv10) with similar tail spikes. Ab31 was able to infect *P. aeruginosa* strain PA14 and two genetically related strains called Tr60 and Tr162, out of 35 diverse strains from cystic fibrosis patients. Analysis of resistant host variants revealed different phenotypes, including induction of pigment and alginate overproduction. Whole genome sequencing of resistant variants highlighted the existence of a large deletion of 234 kbp in two strains, encompassing a cluster of genes required for the production of CupA fimbriae. Stable lysogens formed by Ab31 in strain Tr60, permitted the identification of the insertion site. During colonization of the lung in cystic fibrosis patients, *P. aeruginosa* adapts by modifying its genome. We suggest that bacteriophages such as Ab31 may play an important role in this adaptation by selecting for bacterial characteristics that favor persistence of bacteria in the lung.

## Introduction

Cystic fibrosis (CF) is one of the most common life-threatening, autosomal recessive genetic diseases in Caucasian children. This is due to mutations that occur in a single gene encoding the CF transmembrane regulator. The life expectancy of CF patients is above all related to the development of lung disease: the persistence of abundant mucous secretion in the lungs leads to chronic coughing at a young age, followed by frequent lung infections [1]. The microorganisms that colonize the CF patients’ lungs belong to various bacterial genera. For 30% of CF patients, the predominant bacterial species during early life is *Staphylococcus aureus*, whereas in early adolescence, chronical infection with *Pseudomonas aeruginosa* is common: up to 80% of adult CF patients are colonized by this pathogen [2]. Later during colonization of the lungs, non-motile, anaerobic, mucoid variants of *P. aeruginosa* form a biofilm, a structure that confers resistance to several antimicrobial agents [3]. Usually, the microorganisms account for less than 10% of the dry weight of the biofilm, while 90% is composed of bacterially-produced extracellular polymeric substances (EPS) that form a matrix in which the bacterial cells are embedded [4].

The most abundant component of the EPS produced by *P. aeruginosa* is a polyanionic alginate, a copolymer of mannuronic and glucuronic acids [5]. Typically, *P. aeruginosa* mucoid strains arise in the lungs of CF patients due to mutations in the *mucA* gene or when MucA is degraded by regulated intramembrane proteolysis [6]. Conversion of non-mucoid *P. aeruginosa* strains to mucoid variants can also be the consequence of selective pressure operated by bacteriophages [7], [8]. Recent studies have shown that bacteriophages can drive the emergence of numerous variants with enhanced virulence potential [9], [10].

The majority of *Pseudomonas* tailed phages belong to the order Caudovirales with three main families. Strictly lytic phages are found among the *Myoviridae* with a long contractile tail, and the *Podoviridae* with a short tail, whereas members of the *Siphoviridae* are temperate phages, implying the possibility to undergo lytic or lysogenic interactions with their host [11]. The most striking feature emerging from phage genome comparative analyses is that they are extensively mosaic, with different segments having distinct evolutionary histories. A simple general explanation is that horizontal genetic exchanges play a dominant role in shaping these genome architectures [12], [13]. Gene modules are exchanged using host- or phage-encoded recombination machinery. Although some phages can switch host using different mechanisms, the host preferences represent a significant barrier to genetic exchange. Moreover, phages infecting a common host can also exhibit substantial diversity, creating additional barriers to genetic exchange [12], [13]. Horizontal gene transfer and the pattern of vertical, divergent evolution of phage genomes has led to the definition of different phage genera, and consequently, to a classification based on criteria related to phage genome organization and replication strategy [11]. Despite rapid phage evolution and the short generation time, viral genomes can be stably maintained over ecologically significant time and distance, and this allows their classification. Viral species can be identified and they appear to be globally widespread. Indeed, related members of specific genera with sequence identity up to 99%, can be isolated from different habitats across the globe [14], [15]. The part of the phage genome that varies greatly within each genus is confined to genes encoding the metabolic conversion proteins (early region) and the tail spikes, indicating a local adaptation necessary to infect specific hosts in specific environmental conditions.

In the present study we describe a new phage, vB_PaeP_Tr60_Ab31, whose genome is the result of recombination between two phages belonging to two different families. This phage exerts a selective pressure on *P. aeruginosa,* which could be deleterious to chronically infected patients.

## Materials and Methods

### Ethics Statement

The present project is in compliance with the Helsinki Declaration (Ethical Principles for Medical Research Involving Human Subjects). Strains were collected from sputum as part of the patients' usual care, without any additional sampling. The ethic committee “Comité Consultatif pour la Protection des Personnes dans la Recherche Biomédicale (CCPPRB) Ile-De-France”, who was consulted, specifically approved this study, and declared that patient informed consent was not needed.

### Bacterial Strains

The two reference *P. aeruginosa* strains UCBPP-PA14 [16] and PAO1 [17] were purchased from the “Collection de l'Institut Pasteur” (CIP, Paris, France), and C50 was a gift of U. Römling (Karolinska Institute, Sweden) [18]. The other strains were isolated from sputum of French CF patients, and were previously genotyped using Variable number of tandem repeats (VNTR) analysis (MLVA) [19], [20]. Strains were considered to belong to the same clonal complex when they shared at least 10 VNTR size alleles out of 15. Serological typing was performed using 4 polyvalent and 16 monovalent antisera (Bio-Rad), as described [21]. Briefly, the slide agglutination procedure was performed on 24 h cultures of *P. aeruginosa*: one loop (0.01 ml) of bacterial culture (approximately 3×10^6^ CFU) was mixed with one drop (0.01 ml) of each antiserum (firstly the four polyvalent sera, then the four monovalent sera, corresponding to the positive polyvalent serum). The slide was gently shaken with a rotary movement, and the mixture was examined with the naked eye over a dark surface. A positive reaction was defined as the appearance of agglutination in a maximum of 2 min.

### Phage Amplification and Purification

Phages were amplified on fresh LB agar plates at a ratio of 1 phage for 1000 bacteria. An overnight culture of bacteria grown in LB medium was concentrated 10 times in saline magnesium (SM) phage buffer (50 mM Tris-HCl pH 7.5, 100 mM NaCl, 8.1 mM MgSO_4_, 0.01% gelatin). Phages were added and, after 15 min of incubation at room temperature, the mixture was poured onto a round plate together with 4 ml of soft agar. After complete bacterial lysis (≈ 8 h), 5 ml of SM phage buffer supplemented with a drop of chloroform were added to the recovered soft agar, containing phage particles. After centrifugation, the supernatant was filtered through a 0.22-μm pore size membrane, and kept at 4°C.

### Small Drop and Double Agar Plate Assay

For the small drop assay, 50 μl of 10X concentrated *P. aeruginosa* overnight culture were added to 4 ml molten soft agar (0.7%), and poured over an LB agar plate. Then, 10 μl of serially diluted test lysate were spotted onto the bacterial lawn. For the double agar plaque assay, a mixture of 50 μl of bacterial suspension and 10 μl of phages at ten-fold serial dilutions was kept for 15 min at room temperature, and then poured onto a solid agar plate with 4 ml of soft agar. Plates were inverted and incubated overnight at 37°C.

### Liquid Infection

LB medium (10 ml), supplemented with 10 mM CaCl_2,_ was inoculated at 2.5% with an overnight culture of the indicator strain, and incubated at 37°C. When an OD_600_ of 0.6 was reached, phage suspensions at different multiplicities of infection (M.O.I.) were added. The OD_600_ was periodically measured, and when a significant reduction of the culture density was recorded, 50 μl of chloroform were added in order to facilitate bacterial lysis and release of phages. The suspension was centrifuged at 2,500×*g* for 10 min at 4°C to eliminate bacterial debris, and the supernatant was filtered through a 0.22 μm filter.

### Electron Microscopy Examination

Phage preparations were stained with 2% potassium phosphotungstate (pH 7.0), and then visualized using an EM208S transmission electron microscope (FEI, Eindhoven, The Netherlands) operating at 80 kV.

### Isolation of Resistant Bacteria

Putative resistant bacteria were isolated by simply picking bacterial colonies growing inside the lysis zone of a small drop assay, and streaking them onto new plates. Putative resistant bacteria were also recovered at the end of the liquid infections, by directly streaking 1 μl of the phage-bacterial mixture on a solid agar plate. Up to twenty colonies were picked and challenged with phages through the small drop assay. Some of them were susceptible to phage Ab31, and were thereafter called “non-resistant”. Thermolysates of both resistant and non-resistant strains were prepared by resuspending a colony in 100 μl of water, heating at 95°C for 10 min, followed by cooling on ice for 5 min. Centrifugation was performed at 2,500×*g* for 10 min at 4°C to pellet cell debris, and 2 μl of the supernatant were used for PCR amplification.

### Phage DNA Purification

Phage DNA was purified using a rapid method adapted from [22], as described in [14]. Briefly, phages were amplified on fresh LB agar plates for 8 h at 37°C, then 5 ml of SM buffer were added to the plate, followed by overnight incubation at 4°C. The buffer was transferred to a tube, and bacterial debris were pelleted by centrifugation at 2,500×*g* for 10 min at 4°C. A mixture of 0.2 ml 2 M Tris-HCl pH 7.5, 0.4 ml 0.5 M EDTA, 0.2 ml 10% SDS and 10 μl diethylpyrocarbonate was added to 4 ml of supernatant. Following incubation at 65°C for 30 min, the tube was cooled on ice, and 1 ml of 5 M KOH was added. After 1 h incubation on ice, centrifugation was performed at 25,000×*g* for 20 min at 4°C. DNA contained in the supernatant was precipitated with 2 vol of absolute ethanol, pelleted by centrifugation, washed twice with 70% ethanol, dried and dissolved in 0.4 ml of TE buffer (10 mM Tris-HCl pH 7.5, 1 mM EDTA). Bacterial DNA was purified using the classical CTAB (cetyl-trimethylammonium bromide)-phenol extraction method as described [19]. Purified DNA was resuspended in TE buffer. The quality and concentration of DNA was measured using a ND-1000 Spectrophotometer (NanoDrop®, Labtech, Palaiseau, France).

### Sequencing

Whole genome sequencing was performed by the CNRS sequencing facility in Gif sur Yvette (IMAGIF) using the Illumina platform (Illumina Genome Analyzer IIx). Assembly of short sequence reads was performed using BioNumerics tools (Applied Maths, Sint-Martens-Latem, Belgium) as described [14]. The phage genome was annotated automatically using the BaSyS annotation tools [23]. Bacterial genome contigs were annotated using BioNumerics annotation tools. Detailed methods are available on the website http://bacteriophages.igmors.u-psud.fr.

The annotated Ab31 phage sequence has been deposited at EMBL-EBI under accession number HG798806. Total reads of bacterial genome sequences have been deposited at EMBL-EBI under accession number PRJEB5001.

### PCR Detection of Phage DNA

Oligonucleotides selected to test for the presence of phage DNA in resistant bacteria and to analyze the deletions in bacterial genomes, are listed in Table 1. PCR was performed using purified DNA and Taq polymerase as recommended by the supplier (VWR, Strasbourg France). PCR products were analyzed on 2% agarose gels in 0.5X TBE buffer.

**Table 1:** List of primers used for PCR amplification.

Phage Ab31	
Ab31-Reg1-F	GACTCAGACCACTGAGATGA
Ab31-Reg1-R	ACGTGTTGGCAGTTGTAGAA
Ab31-Term-F	TACAACGCGGATATCCGTGT
Ab31-Term-R	TGCTCCCTCTGATGGACAAA
*P. aeruginosa*	
PaTr60_Del22kb_F	TCATCCACTGTACGCCGCCG
PaTr60_Del22kb_R	CCGTTCCTGATGCTCGACCAGT
PaTr60_Del11kb_F	GACCATGACCTTGTCGCCAT
PaTr60_Del11kb_R	AGGAGGAAATGGGTGCGGAA
Porin1_PaerDel234_F	GAAATAGAGATTGCGCAGGC
Porin1_PaerDel234_R	CACCTTCGACGAGAGACACA
CupA_Paer_F	AGGATCGTCGGCGAGTAGTA
CupA_Paer_R	CTCTATAGCGGCTACTACAC
Porin2_PaerDel234_F	CTCAAGGACATCTACCGACA
Porin2_PaerDel234_R	AAGTCGCCGATCTGGATGAA
PaerDel234_Flank_F	TCCATCGCCTGCATGGCTTC
PaerDel234_Flank_R	CGGCATAACTTCAATCAGGC

## Results

### Ab31 Virulence Spectrum

*P. aeruginosa* phage vB_PaeP_Tr60_Ab31, subsequently called Ab31, was isolated in Abidjan (Ivory Coast) as part of a study to determine the phage diversity in waste water of this city [Essoh *et al.* submitted]. Ab31 was originally enriched on *P. aeruginosa* strain PA14, and subsequently amplified in this strain or in Tr60 (both of serotype O10). A total of 36 *P. aeruginosa* strains were tested for their susceptibility to the phage, including strains from the most frequently encountered clonal complexes in CF patients, PA14 and C50 [24], and reference strain PAO1 (Table 2). Six strains were shown to belong to the PA14 clonal complex (Tr60, Tr162, C7-11, C5-17, C9-12 and C8-12), and seven strains (Tr60, Tr162, C1-3, C3-1, C3-11, C4-14 and C9-5) were of serotype O10. The latter strains were selected in case the host O antigen would serve as a receptor for the phage, as described for *Vibrio cholera* phage VP4 [25]. Ab31 was responsible for complete lysis of Tr60 and Tr162, two non-mucoid strains with the same genotype, isolated from two CF patients at a one year interval, in the same hospital [19]. With the other strains no significant signs of lysis were detected.

**Table 2:** List of the strains used and susceptibility to Ab31.

Strain	Description	Serotype^a^	Source or reference	Ab31 growth^b^
PA14	Sequenced	10	[16]	C+++
PAO1	Sequenced	5	[17]	0
C50	Clone C	UN	[18]	0
Tr60	PA14-clone	10	[19]	C+++
Tr162	PA14-clone	10	[19]	C+++
C7-11	PA14-clone	15	[14], [20]	trace
C5-17	PA14-clone	17	[14], [20]	0
C9-12	PA14-clone	17	[14], [20]	0
C8-12	PA14-clone	6	[14], [20]	trace
C1-1		5	[14], [20]	0
C1-2		3	[14], [20]	0
C1-3		10	[14], [20]	0
C1-11	Mucoid	15	[14], [20]	trace
C1-14		1	[14], [20]	0
C2-10		4	[14], [20]	0
C2-18		12	[14], [20]	0
C3-1		10	[14], [20]	0
C3-2		13	[14], [20]	trace
C3-11		10	[14], [20]	0
C3-15		17	[14], [20]	0
C3-16		1	[14], [20]	0
C3-18		16	[14], [20]	0
C3-19		6	[14], [20]	0
C4-14		10	[14], [20]	0
C5-2		17	[14], [20]	0
C5-13		17	[14], [20]	0
C7-6		3	[14], [20]	0
C8-5		5	[14], [20]	0
C8-7		12	[14], [20]	0
C8-14		1	[14], [20]	0
C8-15	Mucoid	1	[14], [20]	0
C8-20		2	[14], [20]	0
C9-5		10	[14], [20]	0
C9-11		17	[14], [20]	0
C9-17		17	[14], [20]	0
C10-5		UN	[14], [20]	0

a UN, unknown.

b 5 μL of Ab31 stock suspension (≈ 10^8 ^PFU/ml) were spotted on *P. aeruginosa* lawns. C+++, complete clearing; trace, a few individual plaques; 0, a turbid spot where the pipette tip touched the agar.

### Phage Characteristics

**Figure 1:**
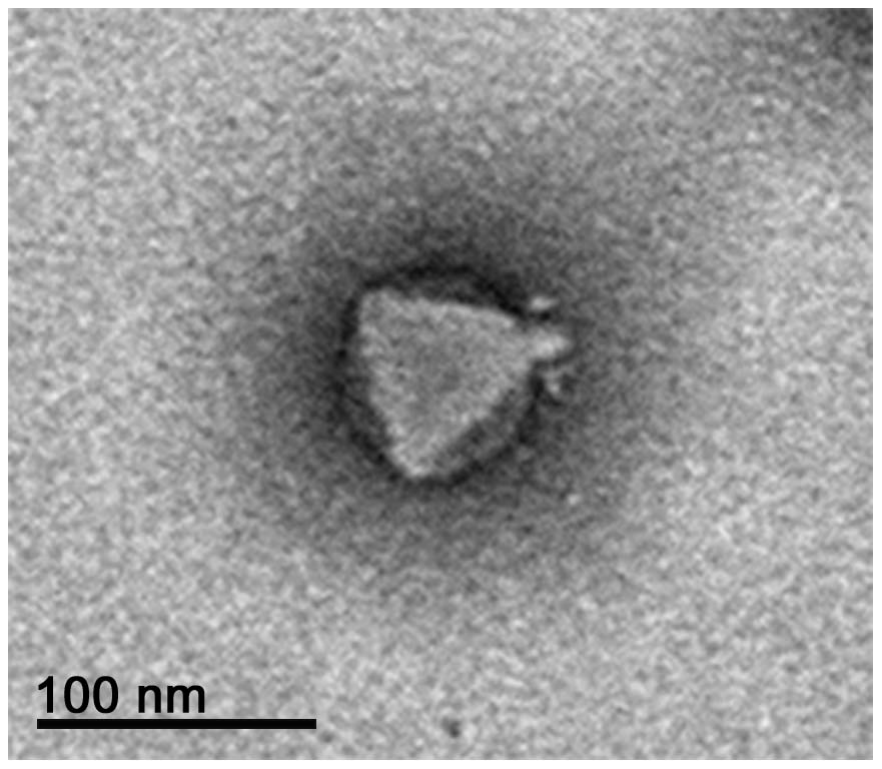
Electron microscopy analysis of phage Ab31. Scale bar represents 100

The morphology of phage Ab31 was determined by transmission electron microscopy (Fig. 1). The phage possesses an icosahedral head with a diameter of approximately 60 nm, and a short non-contractile tail. Moreover, the subterminal tail spikes were similar to those of phage AF of *P. putida* [26].

Infections in solid agar plates and in liquid medium were performed to analyse the Ab31 multiplication characteristics. When a phage suspension was analyzed on indicator strains using the double agar plaque assays, small clear plaques without a halo were observed. Dot assay revealed a clear zone with only a few small resistant colonies. Since it was known that many phages require CaCl_2_ to adsorb on the bacterial surface, the infection in liquid medium was performed using LB supplemented or not with 10 mM of CaCl_2_. Upon infection at an M.O.I. of 0.01, in the presence of 10 mM CaCl_2_, production of PFUs was stimulated 100-fold. In these conditions, the adsorption time was 4 min and the burst size was 30–50 phages per cell.

**Figure 2:**
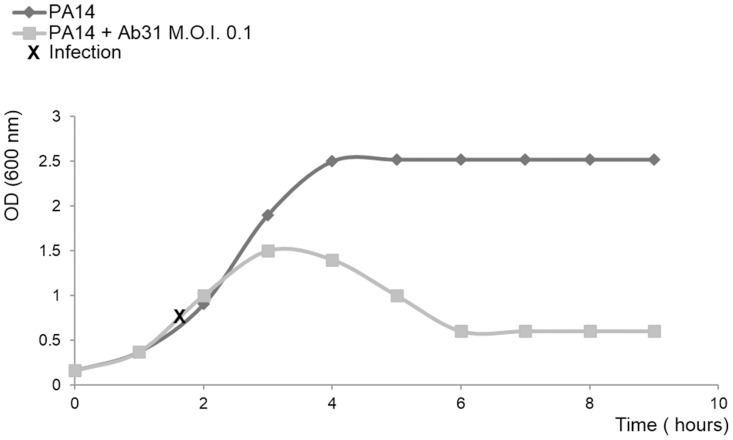
Growth curve of uninfected PA14 (dark grey curve) and of PA14 infected by Ab31 at an M.O.I. of 0.1 (light grey curve).

Infection in liquid LB medium at an M.O.I. of 0.1 never led to a complete clearing of the bacterial culture (Fig. 2), but we observed that not all the bacteria that survived after infection were resistant, when later challenged with Ab31. This resembles the phenomenon of persistence, in which a subset of an isogenic bacterial population occurring within a susceptible population, tolerates antibiotics [27].

Interestingly, we observed that phage infection led to a change in color of the bacterial culture from yellowish-green to green. It is likely that the presence of the phage affects the production of the pyoverdine, a virulence factor of *P. aeruginosa* [28].

### Genome Characteristics

**Figure 3:**
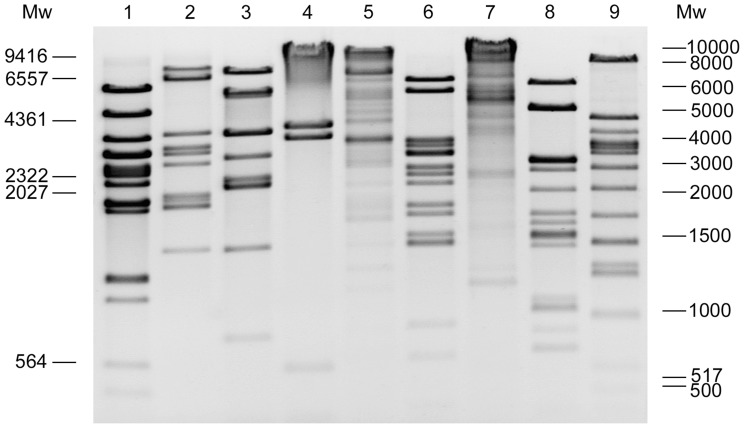
Restriction enzyme analysis of the Ab31 genome. Ab31 phage DNA (2 μg) digested with *Eco*RI (1), *Hind*III (2), *Sma*I (3), *Ssp*I (4), *Not*I (5), *Cla*I (6), *Pvu*II (7), *Sal*I (8) and *Sph*I (9) were analyzed by electrophoresis on a 0.8% agarose gel. On the left the λDNA/*Hind*III and on the right 1 kbp ladder are reported and they were used as molecular weight markers (Mw).

The Ab31 genome encompasses 45,550 bp, and the overall GC-content is ≈ 57%, which is lower than that of the *P. aeruginosa* PA14 genome (66.3% G+C), a characteristic shared by other *P. aeruginosa* phages [29]. Comparing the virtual gel obtained by *in silico* restriction endonuclease analysis with the experimental restriction enzyme banding pattern (Fig. 3), it was possible to establish that Ab31 DNA is apparently circular. Indeed, the number of fragments expected from *in silico* digestion of the circular Ab31 DNA with *Eco*RI, *Hind*III, *Sma*I, *Ssp*I, *Cla*I, *Sal*I and *Sph*I was 17, 12, 12, 6, 18, 23 and 19 respectively, perfectly matched, in number and in size, with the bands obtained by experimental agarose electrophoresis. Ab31 DNA digestion using *Not*I and *Pvu*II, from which 3 and 2 fragments, respectively, were expected using *in silico* digestion analysis, produced many faint bands in addition to the expected ones. This phenomenon, which was observed repeatedly, indicates the existence of a non-specific digestion also called relaxed sequence recognition or star activity [30].

**Figure 4:**
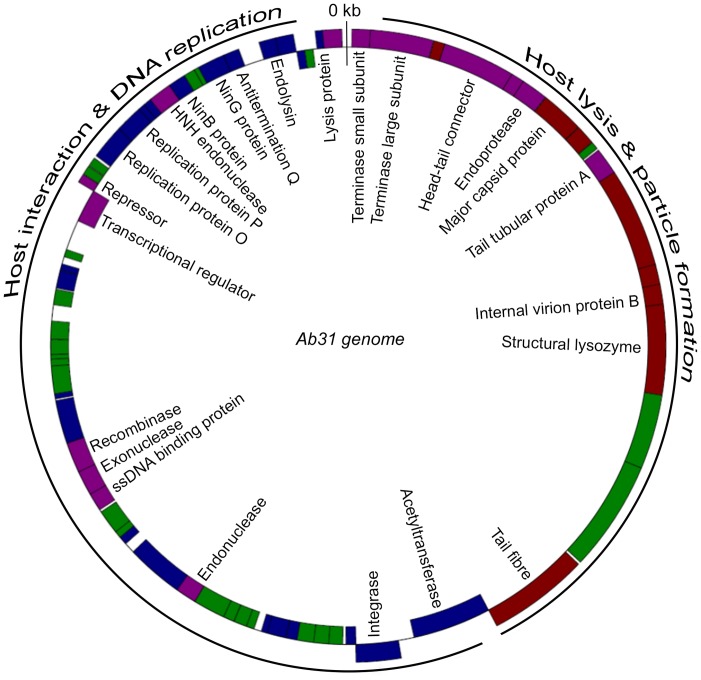
Annotation of the Ab31 genome. The Ab31 hypothetical terminal ends are at the 1 position. The morphogenesis module includes proteins similar to those encoded by phage AF (red), whereas the replication, recombination and lysis modules consist of genes similar to those of phage PAJU2 (blue). Genes encoding hypothetical proteins which have homologies with other phages are shown in purple color. Genes encoding hypothetical proteins of unknown function are shown in green color.

Overall the Ab31 genome did not align with any known phage sequence. However, at the nucleotide level it showed some rare regions of homology with structural genes of phage AF [26] and of a prophage of *P. putida* GB-1 strain [CP000926-1] (up to 70% DNA-DNA similarity), and with genes involved in replication in phage PAJU2 [31] (from 85 to 97%). At the protein level it was possible to observe additional similarities with AF (shown in red on Fig. 4) and PAJU2 (shown in blue in Fig. 4). AF and PAJU2 are two lambdoid phages whose genomes are respectively 42,689 bp and 46,872 bp long. Attempts to localize the genome ends by PCR analysis were not successful, as expected if the genome adopts a circular configuration. Therefore we were not able to determine the position of the first nucleotide, and we decided to assign it by comparison with the related phage AF.

In the Ab31 genome, 69 putative “open reading frames” (ORFs) were identified. Thirty five ORFs were transcribed on the positive strand (Fig. 4). On the basis of sequence similarity comparisons in the GenBank database, 25 ORFs could be assigned to putative functions. The majority of the other ORFs exhibited similarity to uncharacterized bacterial or phage proteins. No tRNA genes were predicted.

Nucleotide position 1 was 60 bp upstream of the ATG codon of a putative protein containing a Helix Turn Helix domain, and sharing similarities with the small terminase subunit encoded by *Escherichia coli* phage phiv10 [32] and *Salmonella enterica* phage epsilon 15 [33]. A putative phage large terminase subunit, encoded immediately downstream of the predicted first coding sequence, shared similarities (maximal identity of 77%, E-value lower than e-200) with a prophage-encoded sequence located within the *S. enterica* serovar Wandsworth str. A4-580 genome. The terminase genes constitute the packaging module typically located at the beginning of the so-called late region of the phage genome. A large part of the late region usually encodes the morphogenesis proteins, whereas genes located in the early region are necessary to initiate the phage multiplication cycle. Following phage adsorption to the bacterial surface, early genes are injected first and, in some phages, they allow complete injection of the phage genome [34]. Phage Ab31 showed high similarity in the late region with the podovirus AF of *P. putida* and the prophage of *P. putida* strain GB-1: indeed, eight coding sequences reported in Fig. 4 resembled those of phage AF. These included the putative major capsid protein (maximal identity of 75%, E-value of 8e-180), internal virion protein B (maximal identity of 35%, E-value of 4e-6), the structural lysozyme (maximal identity of 33%, E-value of 8e-32) and the tail spike protein (maximal identity of 40%, E-value of 4e-37). Moreover, three hypothetical proteins located in the same region showed similarities with hypothetical proteins gp4, gp9 and gp12 of phage AF.

The second major block of genes of the Ab31 genome, downstream of the putative tail spike coding region, constitutes the so-called early/middle region. Twenty-three putative and hypothetical proteins encoded by genes located in this region shared similarities with the *P. aeruginosa* siphovirus PAJU2, showing maximal identity percentages that vary from 39% to 100%. This region starts with a putative acetyl-transferase sharing 46% identity with the PAJU2 acetyl-transferase (E-value lower than e-200), and a putative PAJU2-like integrase (identity 99% with PAJU2; E-value lower than e-200). Other related proteins included putative replication proteins O and P, NinB protein, phage antitermination protein Q and endolysin (Fig. 4).

### Bacterial Resistance

Different conditions were used for infecting bacteria, in order to favor different resistance mechanisms. Most of the resistant and/or lysogenic bacteria were obtained from typical infection at 37°C in liquid medium of *P. aeruginosa* strains PA14, Tr60 and Tr162, at an M.O.I. of 1, 0.1 or 0.01. Some resistant Tr60 bacteria were also recovered from an infection in liquid medium performed at 42°C at an M.O.I. of 1. In this experiment the incubation was prolonged for 72 h, and the phage suspension was added each 24 h at the same M.O.I., for a total of three infections. Ab31-resistant variants of PA14 and Tr162 were also recovered from infections performed on solid agar plates at 37°C or 30°C, using a small drop plaque assay, and extending the incubation for 72 h in order to allow the growth of resistant bacteria inside the lysis zone. With the different approaches, about 80% (91 out of 114) of recovered bacteria were confirmed to be resistant to the phage. A majority of the resistant bacteria obtained from plates formed mucoid colonies, with entire margins and smooth surfaces, a particular characteristic often observed with *P. aeruginosa* strains isolated from sputa of CF patients [Sousa, 2013 #1739]. In contrast, non-resistant bacterial strains derived from the same experiment showed a non-mucoid phenotype and shared the same morphological characteristics with the wild type uninfected bacterial strain. From infected bacteria incubated on plates at 30°C, some mucoid resistant variants showing a brown pigmentation were also isolated. Ten resistant isolates were serotyped to check whether a switch had occurred, but they were all serotype O10 like the parental strains.

**Figure 5:**
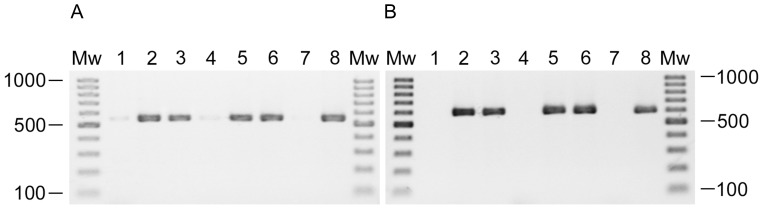
PCR detection of the Ab31 genome in resistant bacteria using Reg1 (A) and Term primers (B). Tr60-E (5) and PA14-P1 (6) are derived from infection on solid agar plates at 37°C and 30°C, respectively. Tr60-10A (2), Tr60-100B (3) and Tr60-100A (4) are derived from an infection assay performed at 37°C in liquid medium at an M.O.I. of 0.1. PA14 (7), Tr60 (1) and Ab31 (8) are used as negative and positive controls, respectively. Samples were run on a 2% agarose gel for 45 min at 135 V. Mw, 100 bp ladder molecular weight markers.

In order to test for the existence of lysogens, the presence of the phage DNA was searched in the Ab31-resistant bacteria after two passages on solid agar medium. Two regions of the phage genome were amplified, designated as Reg1 and Term, and predicted to produce 550 bp and 600 bp long amplicons respectively. The results for some of the resistant isolates and for Ab31 phage DNA used as a control, are shown in Fig. 5. When the DNA of the resistant bacteria was amplified using the Reg1 (Fig. 5A) or Term primers (Fig. 5B), a band of the expected size was detected with every tested isolate except for Tr60-100A. This Ab31-resistant variant was derived from infection in liquid medium at 37°C at an M.O.I. of 0.1. All the resistant variants from solid infection were positive for phage DNA, whereas only 40% of resistant from liquid infection contained phage DNA. The isolates showing a brown pigmentation were not lysogenized.

### Bacterial Genome Sequence Analysis

In order to study the basis of the resistance and to identify the possible integration site of the phage genome in the bacterial chromosome, the original Tr60 strain and four Ab31 resistant variants were chosen for whole genome sequencing. Tr60-10A and Tr60-100B were two putative lysogenic bacteria derived from infection in liquid medium at an M.O.I. of 1 and 0.1, respectively. Tr60-100A was a non-lysogenic isolate derived from infection in liquid medium performed at an M.O.I. of 0.1. PA14-P1 was a putative lysogenic bacteria. Tr60-10A and Tr60-100A showed a mucoid phenotype. The bacterial draft genomes were assembled and partially annotated using *P. aeruginosa* PA14 as a reference [16], [35].

**Figure 6:**
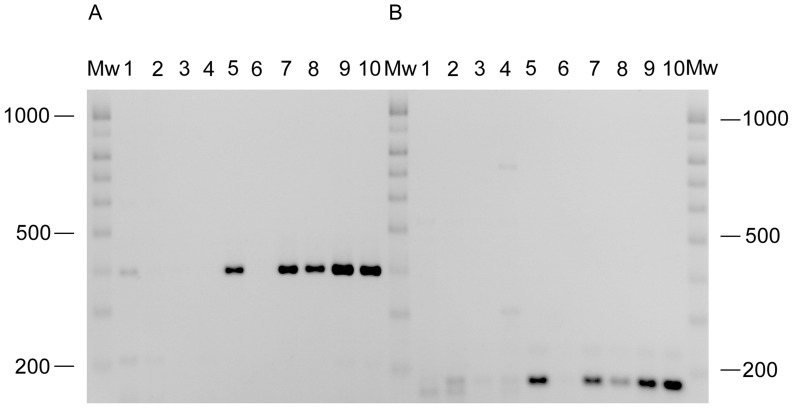
PCR investigation of the 11 kbp (A) and 22 kbp (B) deletions observed in Tr60. C9-12 (1), C5-17 (2), C7-11 (3), C8-12 (4), PAO1 (5), PA14 (6), Tr162 (7), Tr60 (8), Tr60-10A (9), Tr60-100A (10). Samples were run in a 2% agarose gel for 45 min at 135 V. Mw, 100 bp ladder molecular weight markers.

Upon alignment of the sequenced genomes, two deletions were found in the *P. aeruginosa* Tr60 genome as compared to that of PA14. The first deletion encompassed approximately 22 kbp (coordinates 1919495 to 1941370). It started with 2.5 kbp of DNA of unknown function and ended inside a gene encoding a pirin-like protein (ORF PA14_22080 to ORF PA14_22280), and also contained genes for a resolvase and a recombinase. The second deletion encompassed approximately 11 kbp, and covered exactly the sequence of the Pf1 prophage of *P. aeruginosa* PA14 (Genbank: AY324828). Both deletions were identified in Tr60, in all Tr60 variants that were sequenced and in the genome of reference strain PAO1. The existence of the two deletions was confirmed by PCR in Tr60 and its Ab31-resistant variants using primers localised in the flanking regions (Table 1; Fig. 6). The deletions were also observed in Tr162 and in PAO1, as expected. In addition to the two deleted regions, approximately 215 “single nucleotide polymorphisms” (SNPs) were found when the genome sequences of Tr60 and PA14 were compared.

**Figure 7:**
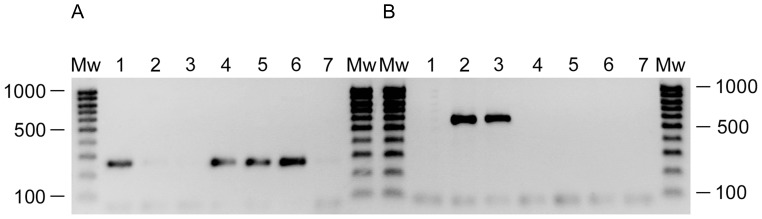
PCR investigation of the 234(A) and Flank234 primers (B). Tr60 (1), Tr60-10A (2), Tr60-100A (3), Tr60-100B (4), PA14-P1 (5), PA14 (6), Negative control (7). The experimental conditions are those of Fig.5.

We then looked at the differences between Tr60 and its Ab31-resistant variants. A deletion of about 234 kbp was found in Tr60-10A and Tr60-100A, two mucoid variants obtained from different infections performed with the same Tr60 bacterial culture. Using *P. aeruginosa* PA14 as a reference genome, 175 coding sequences were found to lie within this region (Table 3, genome coordinates approximately 3190870 and 3424480 in PA14). Some of these coded for enzymes involved in amino acid uptake or biosynthesis, for glucose metabolism and for transmembrane proteins. No homologous genes were found in the other regions of the bacterial genome except for the porins. A cluster of five genes involved in the CupA fimbrial organelle assembly was possibly relevant to phage resistance: the chaperone CupA1, the fimbrial subunit CupA2, the usher CupA3, CupA4, an atypical adhesin, and the chaperone CupA5 [36]. Primer pairs were selected within this region (in two porin-encoding genes and in the *cupA*4 gene), and in the flanking sequences, in order to confirm the existence of the deletion by PCR amplification. When amplification was performed with primers localised inside the region of deletion, an amplicon was observed for all the samples tested except for Tr60-10A and Tr60-100A (the result for *cup*A is shown on Fig. 7A). In contrast, amplification with the Flank234 primers, localised on both sides of the deleted region, produced a 600 bp amplicon only for Tr60-10A and Tr60-100A (Fig. 7B). In order to check whether the deletion was pre-existing in a subpopulation of the Tr60 culture before phage infection, 94 bacterial colonies were picked and PCR was performed on thermolysates with the Flank 234 and porin primers. The results showed that none of the isolates were deleted for the 234 kbp region (data not shown).

**Table 3:** List of coding sequences (CDS) and their position in the 234 kbp deleted region.

CDS	Position	Product
1	87..1418	amino acid permease
2	1482..2921	gamma-aminobutyraldehyde dehydrogenase
3	2967..4220	diaminobutyrate–2-oxoglutarate aminotransferase
4	4267.5229	dehydrogenase
5	- strand (5522..7501)	acetate permease
6	- strand (7558..9828)	acyl-CoA synthetase
7	- strand (9291..10061)	dehydrogenase
8	- strand (10058..11284)	acyl-CoA dehydrogenase
9	- strand (11634..12851)	FadE36, aminoglycoside phosphotransferase
10	12886..14838	propionate catabolism operon regulator
11	- strand (14904..15443)	hypothetical protein
12	- strand (15466..17769)	paraquat-inducible protein B
13	- strand (17762..18619)	paraquat-inducible protein A
14	- strand (19040..20638)	aldehyde dehydrogenase
15	- strand (20752..21747)	hypothetical protein
16	- strand (21775..22950)	hypothetical protein
17	- strand (22981..24408)	MFS transporter
18	- strand (24489..25907)	porin
19	- strand (25912..26925)	4-hydroxythreonine-4-phosphate dehydrogenase
20	- strand (26922..27881)	hypothetical protein
21	- strand (27874..29298)	MFS transporter
22	29301..30479	hypothetical protein
23	30464.32554	hypothetical protein
24	32544..33569	LysR family transcriptional regulator
25	33651..34127	hypothetical protein
26	34342..35232	ABC transporter substrate-binding protein
27	35313..36029	amino acid permease
28	36031..36708	amino acid ABC transporter permease
29	- strand (36724..37608)	hypothetical protein
30	- strand (37734..39329)	signal transduction protein
31	- strand (39418..40800)	dehydrogenase
32	- strand (40640..41758)	hypothetical protein
33	- strand (41808..43745)	TetR family transcriptional regulator
34	- strand (42545..44821)	hydrogen cyanide synthase HcnC
35	- strand (43801..45267)	hydrogen cyanide synthase HcnB
36	- strand (45192..45626)	hydrogen cyanide synthase HcnA
37	45789..47114	adenylate cyclase
38	- strand (47111..47767)	hypothetical protein
39	- strand (48272..49708)	carboxylate-amine ligase
40	48802..51048	hypothetical protein
41	50993..51982	hypothetical protein
42	- strand (51966..52247)	hypothetical protein
43	52256..52957	hypothetical protein
44	- strand (52961..55336)	sensor/response regulator hybrid
45	56396..56686	hypothetical protein
46	- strand (56717..57031)	hypothetical protein
47	- strand (56994..57581)	hypothetical protein
48	- strand (57371..58447)	hypothetical protein
49	- strand (59123..60370)	hypothetical protein
50	- strand (59701..60759)	hypothetical protein
51	- strand (60474..61766)	hypothetical protein
52	- strand (61900..62265)	hypothetical protein
53	62570..64174	glycogen synthase
54	64033..65925	glycosyl hydrolase
55	65918..67972	4-alpha-glucanotransferase
56	67752..70745	maltooligosyl trehalose synthase
57	71060..73210	glycosyl hydrolase
58	76155..77918	cardiolipin synthase 2
59	76625..78910	hypothetical protein
60	- strand (78912..81272)	glycogen branching protein
61	- strand (81107..84409)	trehalose synthase
62	- strand (84420..86534)	hypothetical protein
63	- strand (86558..87439)	KU domain-containing protein
64	- strand (87462..87704)	hypothetical protein
65	- strand (87718..88263)	hypothetical protein
66	- strand (88437..90566)	hydroperoxidase II
67	- strand (90647..90814)	hypothetical protein
68	91301..91711	hypothetical protein
69	- strand (91718..94156)	glycogen phosphorylase
70	- strand (94209..94496)	hypothetical protein
71	94740..94928	hypothetical protein
72	- strand (94948..95991)	short-chain dehydrogenase
73	- strand (95834..96352)	ompetence-damaged protein
74	- strand (96363..96608)	metallothionein
75	- strand (96913..99468)	ATP-dependent DNA ligase
76	- strand (99453..100118)	hypothetical protein
77	99851..100615	hypothetical protein
78	- strand (101068..102432)	transporter
79	- strand (102461..103387)	hypothetical protein
80	- strand (103126..104031)	EAL domain-containing protein
81	- strand (103980..104669)	chaperone CupA5
82	- strand (104683..108297)	fimbrial subunit CupA4
83	- strand (106041..108659)	usher
84	- strand (108643..109476)	chaperone CupA2
85	110882..112108	fimbrial subunit CupA1
86	112535..113170	hypothetical protein
87	- strand (113206..114660)	aldehyde dehydrogenase
88	- strand (114676..116313)	dehydrogenase
89	- strand (116418..117350)	LysR family transcriptional regulator
90	- strand (117387..118523)	hypothetical protein
91	118649.119554	LysR family transcriptional regulator
92	119600..120130	hypothetical protein
93	120147..121259	hypothetical protein
94	- strand (121794..122870)	O6-methylguanine-DNA methyltransferase
95	123062..124045	hypothetical protein
96	- strand (124030..124896)	hypothetical protein
97	- strand (124934..125998)	LysR family transcriptional regulator
98	126034..127413	major facilitator transporter
99	127438..128667	porin
100	128700..129443	LamB/YcsF family protein
101	129529..131091	hypothetical protein
102	131159.131635	hypothetical protein
103	- strand (131654..133426)	thiamine pyrophosphate protein
104	133525..133995	hypothetical protein
105	- strand (134394..136025)	short chain dehydrogenase
106	- strand (135137..136177)	esterase
107	- strand (136077..137552)	flavin-binding monooxygenase
108	137699..138733	AraC family transcriptional regulator
109	138861..139727	hypothetical protein
110	- strand (139729..141048)	transmembrane sensor protein
111	- strand (141294..142682)	MFS transporter
112	- strand (142477..144135)	permease
113	- strand (144849..147995)	TonB-dependent receptor
114	- strand (147592..148680)	hypothetical protein
115	- strand (148424..149845)	hypothetical protein
116	- strand (149077..150093)	hydrolase
117	- strand (150527..152425)	asparagine synthetase, glutamine-hydrolysing
118	- strand (152447..153649)	ring-hydroxylating dioxygenase, large terminal
119	- strand (153923..154405)	leucine-responsive regulatory protein
120	154451..155173	kynurenine formamidase, KynB
121	155177..156427	kynureninase
122	155773..157989	amino acid permease
123	158230..160149	hypothetical protein
124	160165..162093	hypothetical protein
125	- strand (162130..163506)	transcriptional regulator
126	163546..163755	hypothetical protein
127	163932..165599	hypothetical protein
128	- strand (165992..168586)	sensory box protein
129	- strand (168758..171127)	elongation factor G
130	171142..173784	TonB dependent receptor
131	173892..175688	carbamoyl transferase
132	175732..176895	MFS transporter
133	176840..177565	hydrolase
134	176941..178200	hypothetical protein
135	178277..180169	copper resistance protein A
136	179851..181221	copper resistance protein B
137	- strand (181241..182575)	hypothetical protein
138	- strand (182667..183848)	pyridoxal-phosphate dependent protein
139	- strand (183994..185604)	ABC transporter ATP-binding protein
140	- strand (185606..186622)	ABC transporter permease
141	- strand (186624..187697)	peptide ABC transporter permease
142	- strand (187699..189507)	ABC transporter substrate-binding protein
143	- strand (189511..192537)	TonB-dependent receptor
144	- strand (192730..193722)	LysR family transcriptional regulator
145	193773..195158	major facilitator transporter
146	- strand (195165..196064)	DNA-binding transcriptional regulator CynR
147	- strand (197377..198330)	Fe2+-dicitrate sensor, membrane protein
148	- strand (198327..199004)	RNA polymerase sigma factor
149	199037..201157	hypothetical protein
150	203113..204468	hypothetical protein
151	204410..204787	hypothetical protein
152	204854..206836	hypothetical protein
153	- strand (208017..209408)	serine/threonine transporter SstT
154	- strand (209734..211104)	amino acid permease
155	- strand (211261..212637)	glutamine synthetase
156	213047..213901	hypothetical protein
157	213157..214200	hypothetical protein
158	- strand (214325..215800)	hypothetical protein
159	- strand (215925..216731)	hypothetical protein
160	217181..218839	thiamine pyrophosphate protein
161	- strand (218847..220016)	hypothetical protein
162	- strand (219521..220477)	hypothetical protein
163	- strand (220564..222048)	transcriptional regulator
164	222029..222676	hypothetical protein
165	- strand (222751..223581)	hypothetical protein
166	- strand (223074..223547)	transcriptional regulator
167	223638..224072	hypothetical protein
168	223754..225226	bile acid/Na+ symporter family transporter
169	225251..227203	ring-cleaving dioxygenase
170	- strand (225281..226636)	glutathione reductase
171	- strand (227385..228275)	UTP-glucose-1-phosphate uridylyltransferase
172	- strand (228272..229873)	nucleotide sugar dehydrogenase
173	- strand (230015..230575)	transcriptional regulator
174	230812..232002	periplasmic multidrug efflux lipoprotein
175	232018..233901	multidrug efflux protein

Compared to Tr60, Tr60-10A and Tr60-100A showed approximately the same amount of SNPs (respectively 29 and 26). The mutations occurred in the same coding sequences for both resistant bacteria. In particular, mutations were found in a transcriptional regulator, in the NADH dehydrogenase I subunit F, the pyoverdine biosynthesis protein PvcA, phenazine biosynthesis protein PhzD, C32 tRNA thiolase and in an MFS transporter. Specific mutations in a gene for an Usher protein and in a gene for a lipase chaperone, were detected in Tr60-10A and Tr60-100A, respectively. The sequence of the Tr60-100B isolate was compared with that of Tr60, and no SNPs were found.

The PA14-P1 genome showed 129 SNPs as compared to PA14. They affected genes encoding the hemolysin activator, the pyochelin synthase, an RNA methyltransferase, an acetyltransferase, the pyoverdin synthase, and several membrane proteins, including multidrug efflux pumps, type III secretion system proteins and an ABC transporter.

### Search for the Phage Integration Site

**Figure 8:**
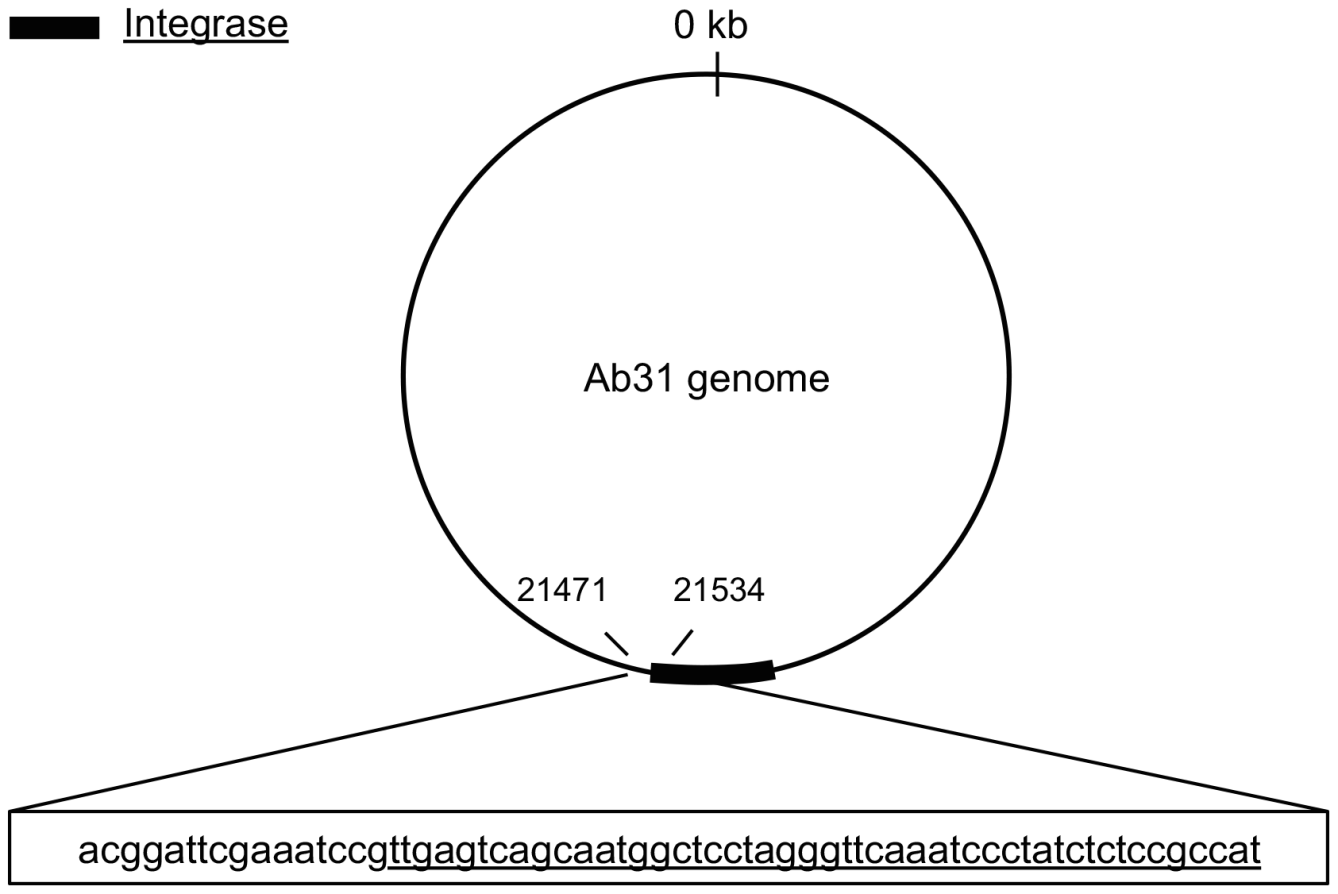
Schematic representation of the Ab31 insertion region. A 64*P. aeruginosa* Tr60 genome. The portion of the shared region that overlaps with the phage integrase encoding gene is underlined.

To identify integrated Ab31 genomes, we first searched for phage reads among the three isolates found to possess phage DNA by PCR, but they were detected only in Tr60-100B. We then looked for phage-bacteria hybrid sequences among the total reads obtained for this isolate. The hybrid reads centered on a 64 bp region found at position 4552973–4553038 in the *P. aeruginosa* PA14 genome (and also in the Tr60 genome), and at position 21471–21534 on the Ab31 genome. These regions correspond in PA14 to the serine tRNA gene (PA14_51230) localized downstream of a glycosyltransferase gene, and, in the Ab31 genome, to a region that covers part of the phage integrase and part of a non-coding sequence upstream of this gene (Fig. 8).

## Discussion

**Figure 9:**
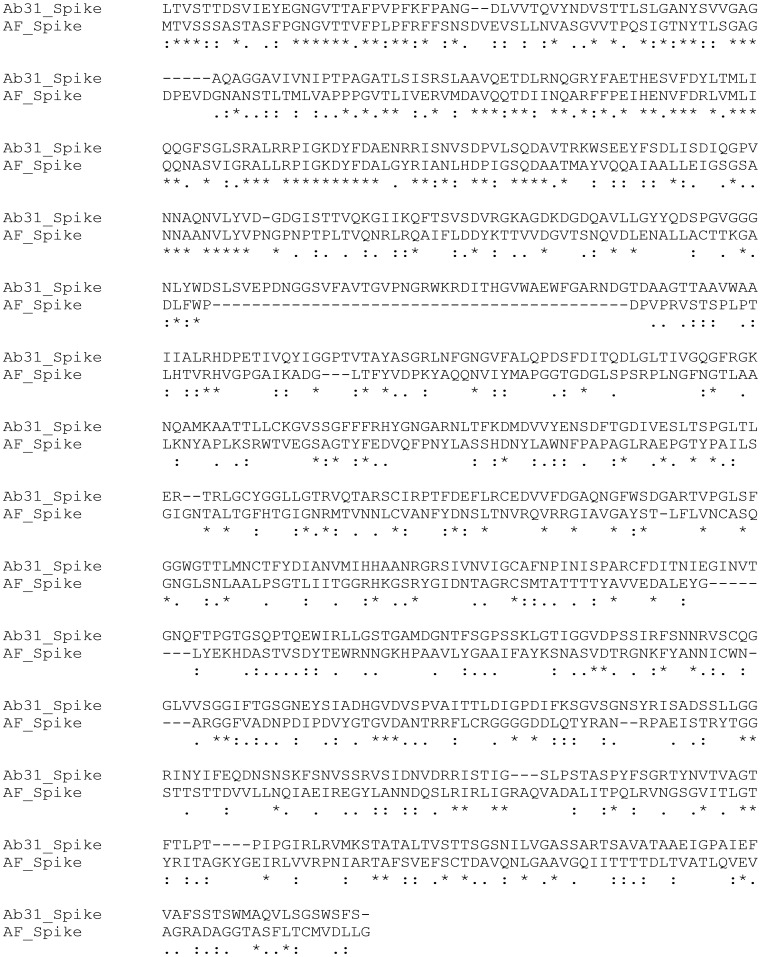
Alignment of the spike protein sequence of phages Ab31 and AF.

Phage Ab31 is a temperate phage genomically related to both the virulent podovirus AF phage of *P. putida* and the temperate siphovirus phage PAJU2 of *P. aeruginosa*. The Ab31 virion structure resembles that of phage AF, *S. enterica* phage Epsilon 15 and *E. coli* phage phiv10, with similar spikes, previously shown to bind and cleave the O-antigen component of the host’s cell surface lipopolysaccharide [37]. Some *Pseudomonas* phages can diffuse through alginate present in *Pseudomonas* biofilms [38] owing to a depolymerizing enzyme that is part of the phage particles. One of the most significant examples of such an activity has been reported for phage AF. A halo surrounds the AF plaques at 30°C, due to an EPS-degrading activity within the tail spikes [26]. We did not see such a halo around Ab31 plaques, whatever the strain used or the temperature. Alignment of the tail spike protein sequences of phage Ab31 and phage AF showed limited homology only at the N-terminus (Fig. 9). Conservation of the N-terminal part is necessary for association of the spikes with the tail structure, whereas the C-terminal part of the spike protein, involved in recognition of and binding to the cell receptor, shows the highest level of variation [39], [40], [41]. This finding provides evidence that bacteria and bacteriophages have co-evolved in order to overcome the barriers that are imposed by one on the other. Recent observations suggest that bacterial resistance to phages in cystic fibrosis patients evolves with the duration of colonization [42]. The infection by Ab31 causes a slight change in the colour of the bacterial culture from yellowish-green to green, as also observed with phage PAJU2 [43]. Mutation in genes for pyoverdine biosynthesis were identified in some resistant isolates, but further analyses are necessary to determine their significance.

Among the diverse strains tested, Ab31 is specific for PA14 and for Tr60 and Tr162, two strains genetically close to PA14, and isolated from French CF patients in the same hospital. Other strains belonging to the same clonal complex but isolated at other locations were found to be resistant to Ab31, as were strains with the same O serotype. Sequencing of Tr60 revealed the presence of two regions of deletion, one of which corresponds to prophage Pf1, also designated Pf5 in the PA14 genome [44]. It was previously shown that Pf4, a Pf1-like prophage in PAO1, mediated the formation of small-colony variants, and was also a factor contributing to the bacterial virulence, but this was not confirmed for Pf5 in PA14 [45], [46]. The 22 kbp region corresponding to the second deletion encodes a resolvase and a recombinase, proteins that usually participate in DNA transfer.

Differences in the bacterial response to phage infection were observed in this study depending on the infection method used. From the infection on solid agar plate, clear lysis zones were observed, an indication that most cells were killed, whereas in liquid culture complete lysis was never obtained. This observation may be related to the formation of pseudolysogens after bacterial infection_._ The genomes of two sequenced Ab31-resistant variants (Tr60-10A and PA14-P1), shown to contain phage DNA when analysed by PCR, surprisingly did not contain phage reads. A possible explanation could be that the phage genome did not integrate in the bacterial chromosome but was retained in a small proportion of cells after infection. Indeed, there is evidence that some of the putative lysogenic bacteria lose the phage genome after several replatings. In contrast, Tr60-100B is a true lysogenic variant. The phage integrated within the bacterial genome through a site-specific recombination process using the shared 64 bp sequence, as shown for PAJU2 [43].

In this study, mucoid variants of *P. aeruginosa* Tr60 and PA14 were obtained after infection of the host in liquid medium. These variants were stably resistant to the phage, although they were not lysogenic. Miller *et al.* showed that temperate phages with elongated heads and flexible tails (similar to PAJU2 virions), induced from CF-associated *P. aeruginosa* strains, were capable of converting non-mucoid strains to the mucoid phenotype [8]. We may hypothesize that the presence of the phage induced a stress that caused mutations in genes involved in alginate production. An alternative explanation could be that the phage selected a subpopulation of mucoid bacteria, with the mucoid layer inhibiting early stages of infection. MucA is a negative regulator of alginate production through sequestration of AlgU, the primary sigma factor responsible for the expression of the alginate biosynthetic operon from the algD promoter [47]. Alternatively, conversion to mucoidy can occur when MucA is degraded by regulated intramembrane proteolysis operated by AlgW [6]. The activation of AlgW, and the consecutive proteolysis of MucA, is thought to be in response to extracellular stress, as well as the accumulation of misfolded envelope proteins. Interestingly, genome sequencing of two non-pigmented mucoid Ab31-resistant variants (Tr60-10A and Tr60-100A) revealed that no modifications of proteins involved in the alginate biosynthesis occurred. Among the Ab31 resistant variants we also obtained brown-colored, mucoid variants of PA14. The pigment which likely corresponds to pyomelanin, accumulated when the plates were kept at room temperature. It was shown that pyomelanin production is due to loss of the homogentisate gene, *HmgA* and this favors persistence in the lung of CF patients [48].

Notably, two mucoid variants of Tr60 carry a large deletion of 234 kbp corresponding to, among others, genes coding for proteins necessary to assemble a fimbrial organelle. This gene cluster which encodes components of the chaperone-usher pathway and a fimbrial unit, participates in biofilm formation [36], [49]. Conceivably fimbriae could be involved in phage adsorption but further investigation is required to confirm or refute this hypothesis. Other deleted genes that might act as phage receptors, are those for two porins, and one being a member of the LamB/YcsF family protein. Previous studies have shown that an outer membrane porin encoded by the *ompLC* gene in *Edwardsiella ictaluri* is required for phage sensitivity [50], while LamB is the receptor for *Escherichia coli* bacteriophage λ. LamB was shown to be sufficient to confer λ phage sensitivity upon transformation of the *lamB* gene into bacteria of different species [51], [52].

Since the two resistant isolates, in addition to the 234 kbp deletion, show a few nucleotide differences (≈ 40 SNPs) with Tr60, two hypotheses could be formulated to explain the origin of the deletion. It is possible that a variant subpopulation with the 234 kbp deletion preexisted in the Tr60 stock suspension, and that the phage infection led to its selection. We could not detect such variants by PCR analysis on 94 isolated colonies or on total DNA extracted from a Tr60 culture. Another possible explanation is that phage infection promoted rearrangement of the host genome. This hypothesis is supported by the finding that at both ends of the 234 kbp region present in the original Tr60 strain, there are sequences of 10 bp in length (ctcggcatga and ctcggcgatga) that differ by a single nucleotide insertion. Notably a similar sequence (c**-**cggcatga) was detected in the phage Ab31 genome at the end of the gene encoding an acetyl-transferase, upstream of the phage integrase. The 10 bp sequence “ctcggcgatga” constitutes the junction of the deleted region on the resistant bacterial genome. Moreover, the sequence upstream the 234 kbp region encodes several proteins involved in transposition, including a bacterial transposase. This suggests that the origin of the deletion in Tr60 was most probably a recombination and/or transposition event in which the phage was also involved. Large genomic deletions have been observed during early stage adaptation of *P. aeruginosa* in CF patients, but none were as large as 234 kbp, which represents about 3.6% of the genome [24], [53]. Rau and colleagues described a deletion of 148 kbp, encompassing the *cupA* cluster [53]. It is not known whether the presence of phages could play a role in the induction of such deletions. Ab31-resistant strain PA14-P1 showed no deletion corresponding to those which characterize Tr60-10A and Tr60-100A. A number of mutations in different genes were observed, but at this time it is impossible to know which one is responsible for phage resistance.

Looking at the Ab31 genome sequence it is possible to distinguish two main modules. The first, showing homologies with the AF phage genome, covers the so-called late region and contains sequences encoding the structural proteins of the phage, such as those for capsid, tail-to-head connector, tail and tail spikes. The second Ab31 genomic region encodes proteins involved in recombination and replication of the phage genome, and constitutes the so-called early/middle region. This contains several genes that show similarities with those of PAJU2 explaining why, although phage Ab31 shows a morphology typical of the virulent AF podovirus, it behaves as a temperate phage capable of lysogenizing *P. aeruginosa* strains. Indeed, the Ab31 insertion site in *P. aeruginosa* is the same as in PAJU2. Phages AF and PAJU2 infect *P. putida* and *P. aeruginosa*, respectively. These bacterial species are closely related, and phage genome exchanges probably occurred during infection of a lysogenic host by the virulent phage. As a result of their mosaic structure, some temperate phage genomes can migrate between unrelated bacteria [54]. Although genetically distant, phages AF and PAJU2 share a lambdoid genome organization which could favor genetic replacement (modular exchanges of gene blocks) [55], [56]. Similar events seem to occur between D3112-like phages morphologically identical to phage lambda, and the transposable coliphage Mu belonging to the *Myoviridae* family [57]. Several types of recombination events are thought to build phage genomes. There are examples of conserved sequences at gene boundaries that could serve to target homologous recombination at these positions, via transposition or site-specific recombination [13]. However a major contributor to phage genome building is illegitimate recombination, or recombination between short conserved sequences (a few bases), coupled with functional selection of genes [13], [58].

## Conclusion

Our observations show that phage Ab31 is the result of a rare recombination event between genomes of two unrelated bacteriophages, normally infecting different bacterial species. It is capable of forming lysogens but its genome can also apparently persists unintegrated for a long time in the bacterial cells, with accompanying repression of virulence functions thus allowing the bacteria to escape lysis. In addition, we show that the phage exerts strong pressure on the bacteria by selecting for variants with new phenotypes, possibly improving their adaptation to chronic lung infection.
